# The multifaceted role of ferroptosis in infection and injury and its nutritional regulation in pigs

**DOI:** 10.1186/s40104-025-01165-1

**Published:** 2025-02-25

**Authors:** Bei Zhou, Junjie Guo, Kan Xiao, Yulan Liu

**Affiliations:** Hubei Key Laboratory of Animal Nutrition and Feed Science, Hubei Collaborative Innovation Center for Animal Nutrition and Feed Safety, Wuhan Polytechnic University, Wuhan, 430023 China

**Keywords:** Ferroptosis, GPX4, Nutritional regulation, Pig

## Abstract

Ferroptosis is a newly identified form of regulated cell death (RCD) characterized by iron overload and excessive lipid peroxidation. To date, numerous studies in human and mouse models have shown that ferroptosis is closely related to tissue damage and various diseases. In recent years, ferroptosis has also been found to play an indispensable and multifaceted role in infection and tissue injury in pigs, and nutritional regulation strategies targeting ferroptosis show great potential. In this review, we summarize the research progress of ferroptosis and its role in infection and tissue injury in pigs. Furthermore, we discuss the existing evidence on ferroptosis regulation by nutrients, aiming to provide valuable insights for future investigation into ferroptosis in pigs and offer a novel perspective for the treatment of infection and injury in pigs.

## Introduction

Cell death can be classified into accidental cell death and regulated cell death (RCD) [1]. RCD is a mechanism by which the host maintains cell function and homeostasis. In addition to apoptosis and autophagy, which have been widely studied, some non-apoptotic RCDs, such as pyroptosis, ferroptosis and necroptosis, have been identified in recent years. Ferroptosis is an iron-dependent form of non-apoptotic cell death with unique metabolic and morphological characteristics and signaling pathways that differ from other cell deaths [2]. Briefly, ferroptosis is characterized by disorder of iron metabolism, overproduction of reactive oxygen species (ROS) and lipid peroxidation, as well as the failure of glutathione peroxidase 4 (GPX4) and System Xc^−^ (a cysteine/glutamate antiporter system) [3, 4]. Morphologically, shrinking mitochondria, ruptured mitochondrial membranes, and decreased mitochondrial crest can be observed in cells undergoing ferroptosis under transmission electron microscopy [5].

In recent years, numerous evidence has demonstrated that ferroptosis plays a significant role in human diseases and organ injuries [6]. Molecular drugs and nutrients that target ferroptosis can effectively mitigate organ damage and prevent the development of diseases [7, 8]. Although ferroptosis in pigs has been poorly studied, current evidence has established a link between ferroptosis and infection and tissue damage of pigs [9, 10]. Here, we review current knowledge regarding the role of ferroptosis in various infection and tissue injury in pigs, and summarize some dietary nutrients that regulate ferroptosis. This review aims to deepen the understanding of the multifaceted role of ferroptosis in injury and disease of pigs, and targeting ferroptosis as a therapeutic strategy by nutritional regulation.

## Characteristics and pathways of ferroptosis

### Dysregulated iron metabolism

**Fig. 1:**
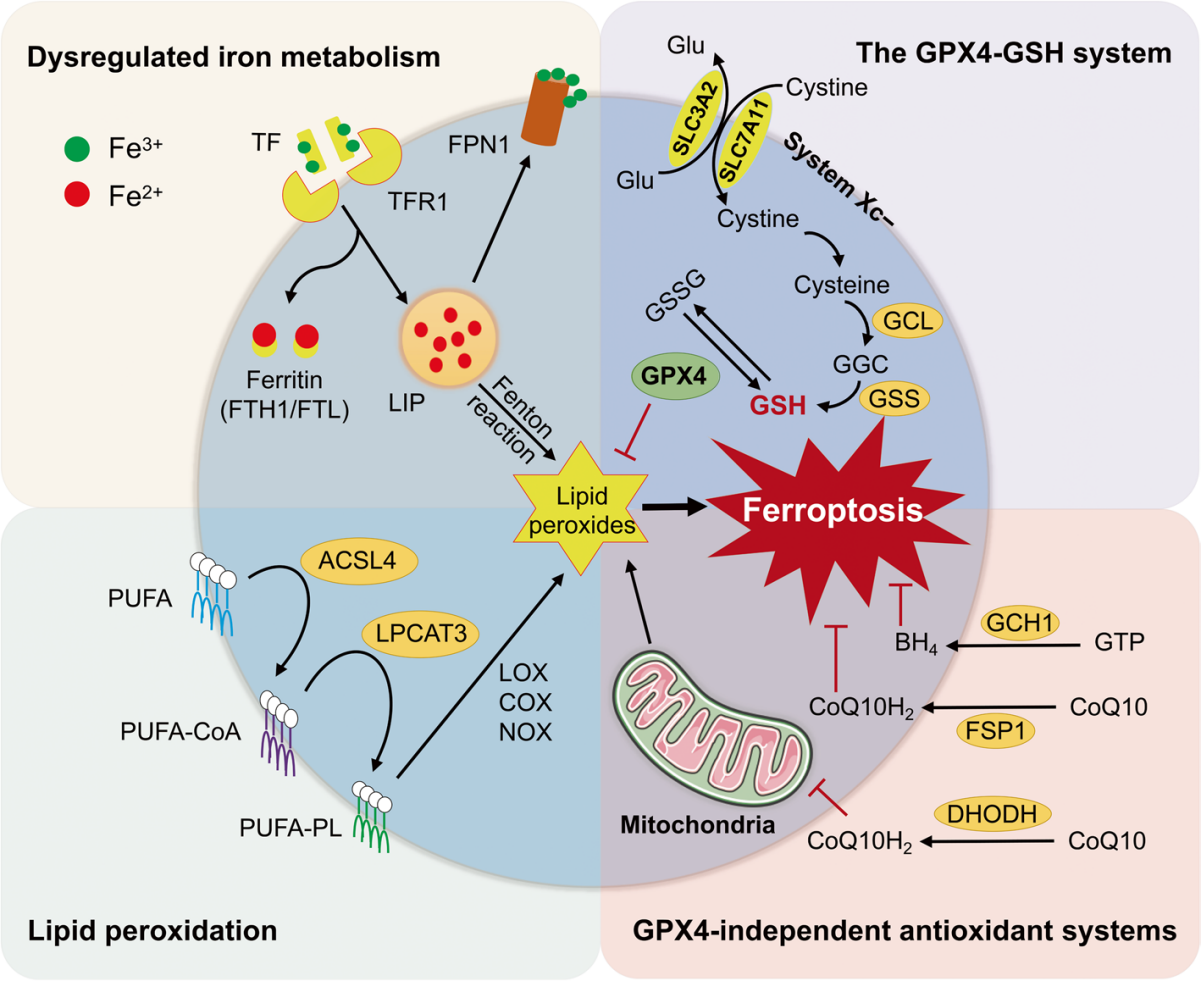
The core regulatory mechanisms and defense systems of ferroptosis. Ferroptosis is characterized by iron-dependent lipid peroxidation and overproduction of ROS. Fe^3+^ binds to TF and is transported to into the cell by TFR1. Ferritin (FTH1, FTL) is the main protein that regulates the storage and utilization of intracellular iron. The excess Fe^2+^ can be oxidized to Fe^3+^ and exported by FPN. Increased iron uptake, decreased iron storage, and inadequate iron efflux can lead to the increase of LIP and subsequent initiation of ferroptosis. The free PUFAs are catalyzed by ACSL4 and LPCAT3 to form PUFA-PLs. In addition, some membrane proteins (such as COXs, NOXs, and LOXs) can accelerate the process of lipid peroxidation. The GPX4-GSH antioxidant system is the main cellular defense mechanism against ferroptosis, which is initiated by System Xc^−^ comprised of SLC3A2 and SLC7A11. Several GPX-independent defense systems have been identified, including the FSP1-CoQH_2_ system, the DHODH-CoQH2 system, and the GCH1-BH_4_ system. ACSL4, acyl-CoA synthetase long-chain family member 4; BH_4_, tetrahydrobiopterin; CoQ10H_2_, ubiquinol; CoQ10, coenzyme Q10; COXs, cyclooxygenases; DHODH, dihydroorotate dehydrogenase; FPN, Ferroportin; FSP1, ferroptosis suppressor protein 1; FTH, ferritin heavy chain; FTL, ferritin light chain; GCL, glutamate-cysteine ligase; GSS, glutathione synthetase; GSSG, oxidized glutathione; GPX4, glutathione peroxidase 4; GCH1, guanosine triphosphate cyclohydrolase 1; GSH, glutathione; LIP, labile iron pool; LOXs, lipoxygenases; LPCAT3, lysophosphatidylcholine acyltransferase 3; NOXs, NADPH oxidases; PUFA, polyunsaturated fatty acid; PUFA-PL, polyunsaturated fatty acid-containing phospholipid; ROS, reactive oxygen species; SLC3A2, solute carrier family 3 member 2; SLC7A11, solute carrier family 7 member 11; TF, transferrin; TFR1, transferrin receptor 1

Ferroptosis is characterized by iron-dependent lipid peroxidation and accumulation of toxic ROS [11] (Fig. 1). Fe^3+^ binds to transferrin (TF) and forms a complex with TF receptor 1 (TFR1), thus entering the cells. The intracellular Fe^3+^ is converted to Fe^2+^ and stored in the labile iron pool (LIP) for later use. Ferritin, which is composed of ferritin heavy chain (FTH) and ferritin light chain (FTL), is the main protein that regulates the storage and utilization of intracellular iron [12]. The excess Fe^2+^ can be oxidized to Fe^3+^ and exported by ferroportin (FPN). Increased iron uptake, decreased iron storage, and inadequate iron efflux can lead to the increase of LIP. Excessive accumulation of Fe^2+^ triggers the Fenton reaction that causes ROS overproduction and lipid peroxidation, and subsequently initiates ferroptosis, which is confirmed by the use of iron chelators and iron supplements [13]. TF and TFR1 have been shown to be the specific markers and key regulators of ferroptosis in recent studies [14, 15].

### Lipid peroxidation

The main feature of ferroptosis is lipid peroxidation, mainly due to the peroxidation of polyunsaturated fatty acid (PUFA) containing bis-allylic carbons [16]. The free PUFAs, including arachidonic acid (AA), are ligated with coenzyme A (CoA) catalyzed by acyl-CoA synthetase long chain family member 4 (ACSL4) [17]. The esterification of PUFA-CoAs is subsequently catalyzed by lysophosphatidylcholine acyltransferase 3 (LPCAT3) to form PUFA-containing phospholipids (PUFA-PLs) [18], which are oxidized to lipid peroxides (PL-PUFA-OOH) and eventually activate ferroptosis. Therefore, suppression of ACSL4 or LPCAT3 expression may be an effective way to inhibit ferroptosis. Current evidence suggests that inhibition of ACSL4 by either pharmacologic inhibitors or gene knockout can prevent ferroptosis, but it seems that knockout of *LPCAT3* does not exhibit the expected blocking effect of ferroptosis [17]. In addition to autoxidation driven by the Fenton reaction [19], the peroxidation of PUFA-PLs can be accelerated by some membrane proteins, such as cyclooxygenases (COXs) [20], NADPH oxidases (NOXs) [21], and lipoxygenases (LOXs) [22]. Toxic lipid peroxides and their decomposition products including malondialdehyde (MDA) and 4-hydroxynonenal (4-HNE) cause rapid and irreversible damage to the cell membrane, ultimately leading to cell death.

### Failure of antioxidant systems

The failure of antioxidant system is one of the important causes of ferroptosis. Among several ferroptosis defense systems that have been identified so far, the GPX4-glutathione (GSH) system is the most critical and has been widely studied. GPX4, the only phospholipid peroxidase, belongs to selenoprotein family that converts PL hydroperoxides to PL alcohols, and thus acts as a core inhibitor of ferroptosis [23]. Genetic or pharmacologic inhibition of GPX4 induces lipid peroxidation and results in ferroptosis in various models [24–26]. GPX4 is regulated by its cofactor GSH, which is synthesized from cysteine, glycine, and glutamate [27]. Importantly, GSH production is dependent on the System Xc^−^, an antiporter on the cell membrane consisting of solute carrier family 3 member 2 (SLC3A2) and solute carrier family 7 member 11 (SLC7A11) [28]. The System Xc^−^ transports cystine into the cell for subsequent GSH production catalyzed by glutamate-cysteine ligase (GCL) and glutathione synthetase (GSS). GSH can be converted to oxidized glutathione (GSSG) catalyzed by GPX4, and conversely GSSG can be reduced to GSH to maintain homeostasis. Previous studies have shown that either cystine deficiency or pharmacologic inhibition of System Xc^−^ (such as erastin, sorafenib, and sulfasalazine) activates ferroptosis. In addition, there are several GPX-independent defense systems, including the ferroptosis suppressor protein-1 (FSP1)-CoQH_2_ system, the dihydroorotate dehydrogenase (DHODH)-CoQH2 system, and the GTP cyclohydroxylase-1 (GCH1)-tetrahydrobiopterin (BH_4_) system [29].

## Ferroptosis and infection

Various types of RCDs have been recognized as defense strategies for host against pathogenic infections. In turn, the pathogens interfere with RCDs to ensure their propagation and survival [30]. Pathogen-induced cell death promotes the release of progeny and contributes to severe inflammation and injury of host [31]. On the other hand, some pathogens can escape host sanction by inhibiting cell death in the early stage of infections, which helps their reproduction [32]. A growing body of evidence suggests the close correlation between ferroptosis and various infections such as viruses, bacteria, or fungi [33]. However, currently only a limited number of studies have reported the occurrence of ferroptosis in viral-infected pigs (Table 1) while ferroptosis caused by prevalent bacterial infections in pigs has not been reported.

**Table 1:** Induction or inhibition of ferroptosis by porcine viruses

Viruses	Effects	Outcomes	Targeted cells	References
PDCoV	Upregulate expression of TFR and LPCAT3; Downregulate expression of ACSL5 and SLC7A11	Activation of ferroptosis inhibits viruse replication	IPEC-J2	[34]
PEDV	Ferroptosis activator (1S,3R)-RSL3 and erastin inhibit System Xc^−^/GPX4 axis and the replication of PEDV	Activation of ferroptosis inhibits viruse replication but does not affect the adhesion, invasion, and release of PEDV	Vero	[35, 36]
SIV	Increase expression of TFR1 and inhibit System Xc^−^/GPX4 axis	Activation of ferroptosis promotes virus replication	A549	[9]
SADS-CoV	Downregulate expression of ACSL4, COX2, and NOX4; Upregulate expression of GPX4, SLC7A11, and FTH1	Inhibition of ferroptosis promotes virus replication	IPI-FX	[37]
ASFV	Do not induce ferroptosis; Brequinar inhibits ASFV replication by activating ferroptosis	Activation of ferroptosis inhibits viruse replication	PAM	[38]

*ACSL* Acyl-CoA synthetase long chain family member, *ASFV* African swine fever virus, *COX* Cyclooxygenase, *FTH* Ferritin heavy chain, *GPX4* Glutathione peroxidase 4, *IPI-FX* Porcine ileum epithelial cell line, *LPCAT3* Lysophosphatidylcholine acyltransferase 3, *NOX* NADPH oxidase, *PAM* Porcine alveolar macrophage, *PDCoV* Porcine deltacoronavirus, *PEDV* Porcine epidemic diarrhea virus, *SADS-CoV* Swine acute diarrhea syndrome coronavirus, *SIV* Swine influenza virus, *SLC7A11* Solute carrier family 7 member 11, *TFR* Transferrin receptor

Porcine viral infectious diseases threaten the global pork industry and public health, which are characterized by rapid spread and widespread prevalence [39, 40]. In general, the host executes a series of defense responses against viral infection, including immune responses and RCDs [41–43]. For example, porcine deltacoronavirus (PDCoV) and porcine epidemic diarrhea virus (PEDV) have been found to activate the ferroptosis signaling pathway, and thus host inhibits virus replication by performing ferroptosis [34, 35]. However, the activation of ferroptosis does not affect the adhesion, invasion, and release of PEDV [35]. It is reported that swine influenza virus (SIV) infection can also cause intercellular iron overload and suppression of system Xc^−^/GPX4 axis activation, and ultimately induce ferroptosis. But unlike PDCoV and PEDV, ferroptosis acts as an aid rather than a blocker in SIV replication and virus-induced inflammatory responses [9]. It is worth noting that SIV-induced ferroptosis is attributed to labile iron accumulation, ROS release as well as lipid peroxidation, while SIV is considered sensitive to ROS and inhibition by them [44, 45]. Besides, the inhibition of PEDV by (1S,3R)-RSL3, a ferroptosis pathway downstream target activator, was observed earlier than its induction of ROS accumulation [36]. Therefore, although the important role of ROS in viral pathogenesis has been widely reported, it seems that the regulation of virus replication by ferroptosis cannot simply be attributed to ROS. In addition to ROS, iron and lipids are also closely related to the impact of ferroptosis on viral replication [46, 47], and the specific mechanism by which ferroptosis affects viral replication needs to be explored. In existing reports about swine viruses, only swine acute diarrhea syndrome coronavirus (SADS-CoV) was found to inhibit ferroptosis during the early stage of infection, thereby favoring its proliferation and survival [37]. Interestingly, the previous study showed that SADS-CoV induced apoptosis in the late stage of infection [48], suggesting that the virus mediates conversion of RCDs in the process of virus replication. Although the current literature on the association between swine virus infection and ferroptosis is very limited, these studies are sufficient to suggest the critical role of ferroptosis in the course of viral infection.

Excitingly, chemical inhibitors or activators targeting ferroptosis can significantly affect viral replication and the associated cell damage and inflammation. Additionally, Brequinar, a highly effective broad-spectrum antiviral molecule, inhibits African swine fever virus (ASFV) replication by activating ferroptosis although ASFV does not induce ferroptosis [38]. Hence, ferroptosis may be a potential target for the interference of viral infection and the development of antiviral drugs. Because of the wide variety of swine viruses and the fact that the same class of viruses show inconsistent effects on ferroptosis in different hosts [37, 49], more comprehensive and systematic studies are needed to be carried out.

## Ferroptosis and injury

A growing body of evidence reveals the important role of ferroptosis in a variety of tissue injuries and non-infectious diseases in humans, such as liver injury, kidney injury, cardiovascular disease, and cancer [50–53]. However, current research has mainly focused on medical studies and mouse models, and less research has been carried out on pigs. Among the existing studies on the involvement of ferroptosis in tissue damage in pigs, the gut and reproductive system are the most concerned (Table 2), while other organs are rarely involved, so here we mainly discuss the studies on these two tissues.

**Table 2:** Ferroptosis involved in gastrointestinal system and reproductive system injury of pigs

Inducers	Objects	Effects	References
Gastrointestinal system			
FAC	IPEC-J2 cells	Reduce cell activity and antioxidant capacity, impair intestinal epithelial barrier, and disrupt mitochondrial function due to the activation of ferroptosis	[54]
DON	Pigs, IPEC-J2 cells	Increase the concentrations of MDA and protein carbonyl in duodenum, jejunum and ileum; Upregulate expression of ferroptotic gene (*DMT1*) and anti-ferroptotic genes (*FTL* and *FTH1*) and downregulate expression of anti-ferroptotic genes (*FPN*, *FSP1* and *CISD1*) in duodenum	[55]
PDCoV	IPEC-J2 cells	Upregulate expression of *TFR* and *LPCAT3*; Downregulate expression of *ACSL5* and *SLC7A11*; Activation of ferroptosis inhibits PDCoV replication	[34]
PEDV	Vero cells	Activation of ferroptosis inhibits PEDV replication	[35, 36]
SADS-CoV	IPI-FX cells	Downregulate expression of ACSL4, COX2, and NOX4; Upregulate expression of GPX4, SLC7A11, and FTH1; Activation of ferroptosis promotes SADS-CoV replication	[37]
Diquat	Pigs	Upregulate expression of *TFR1* and downregulate expression of *GPX4* in jejunum and ileum	[56]
Irradiation	Göttingen minipigs	Downregulate expression of *GPX4* and *SLC7A11* in the intestine	[57]
Reproductive system			
ZEN	Female pigs	Upregulate expression of p-P53, P53, and ACSL4 in the uterus	[58]
Heat stress	Porcine Sertoli cells	Upregulate expression of GPX4, TFR1, and ferritin; Increase level of intracellular ROS	[59]
Zinc	Porcine testis cells	Downregulate expression of SLC7A11, GPX4, and ferritin; Upregulate TF; Increase the concentration of MDA	[60]

*ACSL* Acyl-CoA synthetase long chain family member, *CISD1* CDGSH iron sulfur domain protein 1, *COX* Cyclooxygenase, *DMT1* Divalent metal transporter-1, *DON* Deoxynivalenol, *FAC* Ferric ammonium citrate, *FPN* Ferroportin, *FSP1* Ferroptosis suppressor protein-1, *FTH* Ferritin heavy chain, *FTH* Ferritin light chain, *GPX4* Glutathione peroxidase 4, *LPCAT3* Lysophosphatidylcholine acyltransferase 3, *MDA* Malondialdehyde, *NOX* NADPH oxidase, *PDCoV* Porcine deltacoronavirus, *PEDV* Porcine epidemic diarrhea virus, *ROS* Reactive oxygen species, *SADS-CoV* Swine acute diarrhea syndrome coronavirus, *SLC7A11* Solute carrier family 7 member 11, *TF* Transferrin, *TFR* TF receptor, *ZEN* Zearalenone

### Gastrointestinal system injury

So far, numerous evidence has shown that ferroptosis is involved in intestinal injury induced by a variety of adverse factors, such as iron overload, mycotoxins, and viral infections.

Gut, as the main place of absorption, is more likely to absorb excess iron from diet compared to other organs. Excessive amounts of intracellular iron result in mitochondrial dysfunction and ferroptosis of IECs, as well as intestinal barrier disruption, intestinal microbiota disorder, and increase the susceptibility to pathogen infection. IPEC-J2 cells treated with ferric ammonium citrate (FAC, an iron supplement) showed significant reductions in cell activity and antioxidant capacity, impairment of intestinal epithelial barrier, and disruption of mitochondrial function due to iron overload-induced ferroptosis [54]. Gu et al. [61] reported that the iron overload induced the colitis in mice by modulating ferroptosis and altered the microbiome composition in the feces.

Mycotoxin is another toxic substance easily absorbed by the intestinal from diet, which is more likely to cause intestinal damage in pigs than in other species. Liu et al. [55] reported that diet with deoxynivalenol (DON) at doses of 1.0 and 3.0 mg/kg increased oxidative stress markers MDA in the duodenum, jejunum and ileum of piglets, while ferroptotic gene (*DMT1*) was upregulated and anti-ferroptotic genes (*FPN*, *FSP1* and CDGSH iron sulfur domain protein 1 (*CISD1*)) were downregulated. Furthermore, DON-induced damage in IEPC-J2 cells was mitigated by ferroptotic inhibitor deferiprone, confirming the role of ferroptosis in the gastrointestinal toxicity of DON. Apart from DON, the toxicological mechanisms of many other mycotoxins such as patulin, HT-2 toxin, aflatoxin B1 and zearalenone (ZEN) have been revealed to be closely related to ferroptosis in mouse model. However, direct evidence is lacking on whether enterotoxicity of mycotoxins other than DON to pigs is involved in ferroptosis. Therefore, relevant studies are worth exploring due to the prevalence of mycotoxins in pig feed.

Recent research has revealed that porcine viruses interact closely with their hosts via the ferroptosis pathway. Among the swine viruses targeting gut, PDCoV, SADS-CoV, and PEDV have been reported to activate or inhibit ferroptosis, and chemical inhibitors or activators aimed ferroptosis can regulate virus replication and virus-induced intestinal damage [34, 36, 37].

Moreover, ferroptosis is also present in some intestinal pathological models. Previous studies in our laboratory showed that weaned piglets with intestinal oxidative damage induced by diquat significantly increased expression of *TFR1* and decreased expression of *GPX4* in jejunum and ileum, indicating that diquat activates the ferroptosis signaling pathway in IECs of piglets [56]. Kong et al. [57] reported that irradiation induced the downregulation of GPX4 and SLC7A11 in the intestine in the Göttingen minipig model of hematopoietic-acute radiation syndrome (H-ARS), which was consistent with conditions favoring ferroptosis.

### Reproductive system injury

At present, the adverse factors that have been reported to activate ferroptosis in pig reproductive system mainly include mycotoxins, heat stress, and zinc (Zn) overload.

Mycotoxins not only cause serious damage to the gastrointestinal system of pigs, but also to the reproductive system. Fu et al. [58] revealed that ZEN-induced reproductive toxicity was due to the activation of ferroptosis in female pigs through RNA-seq analysis. Furthermore, it is confirmed that ZEN induced oxidative stress and ferroptosis in a glutathione-dependent manner and could be mitigated by melatonin supplementation in cell model and mouse model. However, up to date, it has not been reported whether other mycotoxin-induced damage to the reproductive system of pigs involves ferroptosis. Notably, in mouse models, a variety of mycotoxins have been reported to cause reproductive system toxicity by the activation of ferroptosis [62]. Although mice are excellent pathological models, similar studies in pigs are necessary.

Recently, Yang et al. [59] reported that heat stress reduced the protein expression of GPX4, TFR1, and Ferritin while increased the level of intracellular ROS in porcine Sertoli cells, which was consistent with the characteristics of ferroptosis, and the decline in cell vitality could be alleviated by Ferrostatin-1. These results confirmed the role of ferroptosis in heat stress-induced injury of porcine Sertoli cells. Mechanistically, heat stress significantly increased the expression of cytochrome P450 cyclooxygenase 2C9 and the content of epoxyeicosatrienoic acids, thus triggering the Ras-JNK signaling pathway and ultimately leading to the activation of ferroptosis of porcine Sertoli cells.

Zn is a critical microelement for physiological process, but excess Zn exposure can lead to testicular dysfunction. Recent study by Li et al. [60] reveals that ferroptosis is partly responsible for the reproductive toxicity caused by Zn overload, and Zn-induced ferroptosis in porcine testis cells is attributed to mitophagy.

In addition, ferroptosis may also be involved in embryonic development in pigs. FSP1 is a glutathione-independent ferroptosis inhibitory factor, which plays a crucial role in the regulation of mitochondrial function and ferroptosis. It has recently been reported to be involved in regulating the porcine early embryonic development and quality. Specifically, inhibition of FSP1 can impair blastocyst formation, lead to mitochondrial dysfunction and ferroptosis, and therefore impairing the quality of porcine early embryos [63].

## Ferroptosis regulation by nutrients

According to the previous description, ferroptosis is closely related to dysregulated iron metabolism and lipid peroxidation. At present, there are few studies on the regulation of ferroptosis by nutrients, especially in livestock and poultry. Existing studies have shown that some nutrients or plant extracts with antioxidant effect play an important role in the regulation of ferroptosis (Table 3). Although only a few of these studies involve pigs, they can still provide references for us to regulate ferroptosis caused by various adverse factors in pig production by nutrients.

**Table 3:** Ferroptosis regulation by nutrients in pigs and some medical/mice models

Nutrients	Models	Mechanisms	References
Selenium	DON-induced intestinal injury in mice	Increase levels of *GPX4* and *4-HNE* by activating PI3K/AKT pathway	[64]
Selenium	Cerebral I/R injury in MCAO model mice and OGD/R model of N2a cells	Upregulate expression of *Mfn1* to alleviate oxidative stress and ferroptosis by promoting mitochondrial fusion	[65]
Selenium	Autism spectrum disorder model mice	Inhibit ferroptosis by regulating Nrf2/GPX4 pathway	[66]
Selenium	Hemorrhagic stroke model mice and HT22 murine hippocampal cells	Augment the transcription of *GPX4* via coordinated activation of the transcription factors TFAP2c and Sp1	[67]
Glycine	Diquat-induced intestinal injury in pigs	Upregulate expression of *SLC7A11* and *GPX4*, and downregulate expression of *TFR1* in ileum	[68]
Glycine	Diquat-induced hepatic injury in pigs	Downregulate expression of *TFR1*	[69]
L-citrulline	Iron overload-induced intestine injury in mice and IPEC-J2 cells	Downregulate expression of *TFR*, *FTH*, and *NCOA4*; Improve oxidative stress by regulating AMPK signaling pathway	[54]
Fish skin gelatin peptides and Gly-Pro-Ala peptide	DON-induced toxicity in mice and IPEC-J2 cells	Inhibit ROS and MDA production and enhance antioxidant enzyme activity by promoting Nrf2 migration	[70]
Lentil peptides	Anemic Caco-2 cells	Downregulate expression of *DMT1* and *TFR*	[71]
Vitamin E	T cell-specific Gpx4-deficient mice with acute lymphocytic choriomeningitis virus and Leishmania major parasite infections	Upregulate expression of *GPX4*	[72]
Vitamin E	Mice	Deplete liver iron stores by suppressing Nrf2 and enhance iron efflux by upregulating expression of liver FPN	[73]
Vitamin E	Mice with conditional deletion of Gpx4 in hepatocytes along with lacking Txnrd1 and selenocysteine tRNA in hepatocytes	Inhibit lipid peroxidation	[74]
Holly polyphenols	Diquat-induced hepatic and intestinal injury in pigs	Upregulate expression of *GPX4* and *SLC7A11* and downregulate expression of *TFR*	[56, 75]
Hesperidin	DON-induced intestinal injury in pigs	Exert protective effects on intestinal epithelium barrier and mitochondria via inhibiting ER-mitochondrial calcium transfer mediated by IP3Rs	[76]
Quercetin	DON-induced intestinal injury in mice	Decrease the levels of TFR, ACSL4, and 4-HNE, and increase the expression of *FTH1*, *SLC7A11*, *GPX4*, *FPN1*, and *FSP1*	[77]
Resveratrol	Intestinal I/R mice model and Caco-2 hypoxia-reoxygenation model	Activate SIRT3/FoxO3a pathway, increase the expression of SOD2 and catalase, and inhibit ROS generation	[78]
Resveratrol	DON-exposed HepG2 cells	Activate SLC7A11-GSH-GPX4 signaling pathway	[79]
Lycopene	Mycotoxins (ZEN + DON + AFB1)-induced intestinal injury in mice	Downregulate expression of *TFR1*, *FTH1*, and *SLC3A2*	[80]
Lycopene	Atrazine-induced hippocampus injury in mice	Upregulate expression of *Nrf2* and *SLC7A11*	[81]
Glycyrrhetinic acid	DON-induced hepatic damage in mice and AML12 cells	Inhibit NCOA4 signaling pathway	[82]
Epigallocatechin gallate	Iron overload-induced hepatic damage in mice	Elevate antioxidant capacity by increasing Nfr2 and GPX4 expression and attenuate iron metabolism disorders by upregulating FTH and FTL expression	[83]
Astragalus polysaccharide	DSS-induced colitis in mice, and RSL3-stimulated Caco-2 cells	Decrease expression of *FTH* and *FTL* and the levels of MDA, GSH, and iron load via inhibiting Nrf2/HO-1 pathway	[84]

*ACSL* Acyl-CoA synthetase long chain family member, *AFB1* Aflatoxin B1, *AMPK* AMP-activated protein kinase, *DMT1* Divalent metal transporter-1, *DON* Deoxynivalenol, *FPN* Ferroportin, *FSP1* Ferroptosis suppressor protein-1, *FTH* Ferritin heavy chain, *FTH* Ferritin light chain, *GPX4* Glutathione peroxidase 4, *GSH* Glutathione, *HNE* Hydroxynonenal, *I/R* Ischemia–reperfusion, *MCAO* Middle cerebral artery occlusion, *MDA* Malondialdehyde, *MFN1* Mito-fusion 1, *NCOA4* Nuclear receptor coactivator 4, *OGD/R* Oxygen–glucose deprivation and reoxygenation, *ROS* Reactive oxygen species, *SIRT3* Recombinant sirtuin 3, *SLC7A11* Solute carrier family 7 member 11, *SOD* Superoxide dismutase, *TFR* Transferrin receptor, *ZEN* Zearalenone

### Selenium

Ferroptosis is mainly regulated by selenium (Se)-dependent pathway [85]. It is regulated by Se mainly due to GPX4, a unique selenoprotein that functions as a phospholipid peroxidase, thereby protecting cells from lipid peroxidation and ferroptosis. Dietary supplementation of Se is an effective strategy to increase intracellular selenium concentration and thus protect cells from ferroptosis. However, relevant studies are currently mainly focused on mouse models and cell models, and direct evidence is lacking in pigs. Fan et al. [64] revealed that dietary supplementation of Se increased the expression of GPX4, p-PI3K, and AKT, and decreased the level of 4-HNE in mice with DON-induced intestinal injury, inhibited DON-induced ferroptosis and thus improved intestinal barrier function. Se treatment apparently attenuated oxidative stress and inhibited iron accumulation in animal model and cell model of cerebral ischemia–reperfusion (I/R) injury [65]. Se mitigated the impairments in the nervous system of mice due to inhibition of ferroptosis by regulating the Nrf2/GPX4 pathway [66]. In addition to being a component of GPX4 synthesis, selenium can also augment the transcription of GPX4 via coordinated activation of the transcription factors TFAP2c and SP1 and thus alleviate haemorrhagic or ischaemic stroke [67]. Similarly, Se increased the expression of GPX4 and the transcription factors TFAP2c and SP1, and ameliorated histological and functional impairment caused by cadmium-induced ferroptosis in sheep kidney [86].

### Amino acids and bioactive peptides

Glycine is one of the components used for the synthesis of endogenous antioxidants GSH, which is the cofactor of GPX4. The antioxidant effect of glycine has been widely reported. Previous studies in our laboratory reveals that dietary glycine supplementation can inhibit the occurrence of cell ferroptosis, thus effectively alleviating the liver and intestinal damage caused by diquat in weaned piglets [68, 69]. Glycine has also been reported to regulate ROS-induced lipid metabolism and further reduce ferroptosis, thereby promoting porcine oocyte maturation and early embryonic development [87]. Glutamate is also necessary to GSH synthesis, but supplementation with glutamate does not appear to inhibit ferroptosis. Instead, high concentrations of glutamate interfere cystine uptake by inhibiting the System Xc^−^, leading to intracellular glutathione depletion and resulting in ROS accumulation, and ultimately activating ferroptosis and inducing neuroexcitotoxicity [88]. Another antioxidant amino acid, L-citrulline, has been shown to have a variety of health benefits, such as improving immune system function, regulating blood sugar levels, and preventing cardiovascular disease. L-citrulline has recently been found to regulate iron metabolism and restrain ferroptosis, improve mitochondrial quality, and improve gut microbiota in mouse model, thereby alleviating intestinal damage caused by iron overload [54]. Mechanistically, L-citrulline activates AMPK pathway in IPEC-J2 cell model, thereby exerting antioxidation and protecting cells from oxidative stress damage.

In addition, some bioactive peptides have been reported to regulate ferroptosis by improving oxidative stress and regulating iron metabolism. For instance, fish skin gelatin peptides and Gly-Pro-Ala (GPA) peptide increase the expression of GSH and GPX4 by activating Nrf2, alleviating the toxicity and oxidative stress induced by DON in the mice and IPEC-J2 cells [70]. Lentil peptides derived protein reduces the mRNA levels of DMT1 and TFR, and increases iron bioavailability, suggesting its regulation of ferroptosis and the improvement of iron deficiency anemia [71].

### Vitamin E

Vitamin E is well known for its excellent ability to scour free radicals and block lipid oxidation, making it one of the most effective and safe natural ferroptosis inhibitors. In several dietary vitamin E, tocopherol is more easily absorbed and utilized by the body, thus α-tocopherol is considered to be the main form of antioxidant activity of vitamin E in the body [89]. But tocotrienols appear to be more effective than tocopherols in protecting cells from ferroptosis because of their superior ability to inhibit LOXs, the key enzymes that regulate lipid peroxidation and subsequent ferroptosis [90]. Dietary supplementation of vitamin E has a promising effect in blocking ferroptosis. Although this has not been reported in pigs, the results in mice and cell models are supportive. Matsushita et al. [72] found that dietary supplementation of high dosage of vitamin E repaired the dysfunction of T cells due to Gpx4 deficiency and protected from acute lymphocytic choriomeningitis virus and Leishmania major parasite infections. Dietary supplementation of vitamin E in mice can improve iron-mediated oxidative damage to the liver by inhibiting the iron- and redox-sensing transcription factor Nrf2, enhancing liver iron efflux and restricting ferroptosis [73]. In addition, dietary supplementation of vitamin E during gestation improves fetal lethality caused by Gpx4 deficiency in mice [74]. This suggests that nutritional regulation of ferroptosis can be carried out during pregnancy to prevent ferroptosis-related diseases earlier.

### Plant extracts

In recent years, many plant extracts, especially polyphenols and flavonoids, are well known for their anti-inflammatory and antioxidant properties [91, 92]. This suggests that plant extracts can be used to regulate ferroptosis. Our laboratory reported that holly polyphenols sourced from *Ilex latifolia* Thunb inhibited the activation of ferroptosis induced by diquat, thus alleviating the histological and functional injury of liver and intestine in weanling piglets [56, 75]. Li et al. [76] found that hesperidin, one of the major flavonoids in citrus fruits that has various biological activities, alleviated mitochondrial dysfunction and ferroptosis in the intestine of piglets exposed to DON. Actually, only the above studies have reported the regulation of ferroptosis by plant extracts in pigs. However, a variety of plant extracts have been found to regulate ferroptosis in medical models or in vitro experiments, which can also provide valuable references for the research and application in pigs. For example, quercetin, a plant polyphenol with anti-inflammatory and antioxidant properties, was reported to reverse DON-induced intestinal oxidative stress and ferroptosis in mouse model [77]. Another polyphenol, resveratrol, is a bioactive ingredient in wine and grape juice. It is an antitoxin secreted by plants under adversity or pathogen attack, and has subsequently been found to have a variety of beneficial effects such as immune regulation and anti-aging, and can be used as a dietary supplement for human health care and disease prevention. It can activate SIRT3 and GSH/GPX4 signaling to relieve ferroptosis of intestinal cells in I/R mice, and restrict DON-induced ferroptosis by activating SLC7A11-GSH-GPX4 signaling pathways in vitro [78, 79]. Lycopene, a carotenoid found in tomatoes, watermelons and other plant foods. It was recently reported to protect the intestine and nervous system by inhibiting mitochondrial damage and ferroptosis in mouse model [80, 81]. Glycyrrhetinic acid is the prominent constituent of glycyrrhize glabra, which has anti-inflammatory, anti-allergic and anti-bacterial effects. It inhibits nuclear receptor coactivator 4 (NCOA4)-mediated ferritinophagy and subsequent ferroptosis, as well as improving mitochondrial function [82]. Besides, epigallocatechin gallate and astragalus polysaccharide have also been confirmed to inhibit ferroptosis in mice [83, 84].

## Conclusions and perspectives

The role of ferroptosis in injury and disease is multifaceted. It can be a strategy for host to maintain normal cell function and homeostasis, and can in turn be hijacked by viruses to promote viral proliferation. There is no doubt that the role of ferroptosis in porcine virus-induced injuries and other non-infectious injuries should not be ignored, although the relevant studies are still insufficient. Importantly, the effect of nutrients on ferroptosis in a large number of medical models is encouraging, which will have important implications for us to use nutrients to prevent and treat various diseases in pig production. Therefore, more comprehensive and in-depth studies on the mechanism of ferroptosis and its regulation in pigs are needed.

## Data Availability

Not applicable.

## Abbreviations

AA: Arachidonic acid
ACSL4: Acyl-CoA synthetase long chain family member 4
AMPK: AMP-dependent protein kinase
ASFV: African swine fever virus
BH_4_: Tetrahydrobiopterin
CISD1: CDGSH iron-sulfur domain-containing protein 1
CoA: Coenzyme A
CoQH_2_: Ubiquinol
COX: Cyclooxygenase
DHODH: Dihydroorotate dehydrogenase
DMT1: Divalent metal transporter-1
DON: Deoxynivalenol
FAC: Ferric ammonium citrate
FPN: Ferroportin
FSP1: Ferroptosis suppressor protein-1
FTH: Ferritin heavy chain
FTL: Ferritin light chain
GCH1: GTP cyclohydroxylase-1
GPX4: Glutathione peroxidase 4
GSH: GPX4-glutathione
GCL: Glutamate-cysteine ligase
GSS: Glutathione synthetase
GSSG: Oxidized glutathione
H-ARS: Hematopoietic-acute radiation syndrome
HNE: Hydroxynonenal
I/R: Ischemia-reperfusion
IEC: Intestinal epithelial cell
LIP: Labile iron pool
LOX: Lipoxygenase
LPCAT3: Lysophosphatidylcholine acyltransferase 3
MDA: Malondialdehyde
NCOA4: Nuclear receptor coactivator 4
NOX: NADPH oxidase
PDCoV: Porcine deltacoronavirus
PEDV: Porcine epidemic diarrhea virus
PL: Phospholipids
PUFA: Polyunsaturated fatty acid
RCD: Regulated cell death
ROS: Reactive oxygen species
SADS-CoV: Swine acute diarrhea syndrome coronavirus
Se: Selenium
SIRT3: Silent information regulator 3
SIV: Swine influenza virus
SLC3A2: Solute carrier family 3 member 2
SLC7A11: Solute carrier family 7 member 11
TF: Transferrin
TFR1: TF receptor 1
ZEN: Zearalenone
Zn: Zinc
